# Experimental and mathematical characterization of coronary polyamide-12 balloon catheter membranes

**DOI:** 10.1371/journal.pone.0234340

**Published:** 2020-06-24

**Authors:** Markus A. Geith, Jakob D. Eckmann, Daniel Ch. Haspinger, Emmanouil Agrafiotis, Dominik Maier, Patrick Szabo, Gerhard Sommer, Thomas G. Schratzenstaller, Gerhard A. Holzapfel

**Affiliations:** 1 Faculty of Computer Science and Biomedical Engineering, Institute of Biomechanics, Graz University of Technology, Graz, Austria; 2 Biomedical Engineering Department, King’s College London, London, United Kingdom; 3 Medical Device Laboratory, Regensburg Center of Biomedical Engineering, Technical University of Applied Sciences Regensburg, Regensburg, Germany; 4 Department of Structural Engineering, Norwegian University of Science and Technology (NTNU), Trondheim, Norway; Politecnico di Milano, ITALY

## Abstract

The experimental quantification and modeling of the multiaxial mechanical response of polymer membranes of coronary balloon catheters have not yet been carried out. Due to the lack of insights, it is not shown whether isotropic material models can describe the material response of balloon catheter membranes expanded with nominal or higher, supra-nominal pressures. Therefore, for the first time, specimens of commercial polyamide-12 balloon catheters membranes were investigated during uniaxial and biaxial loading scenarios. Furthermore, the influence of kinematic effects on the material response was observed by comparing results from quasi-static and dynamic biaxial extension tests. Novel clamping techniques are described, which allow to test even tiny specimens taken from the balloon membranes. The results of this study reveal the semi-compliant, nonlinear, and viscoelastic character of polyamide-12 balloon catheter membranes. Above nominal pressure, the membranes show a pronounced anisotropic mechanical behavior with a stiffer response in the circumferential direction. The anisotropic feature intensifies with an increasing strain-rate. A modified polynomial model was applied to represent the realistic mechanical response of the balloon catheter membranes during dynamic biaxial extension tests. This study also includes a compact set of constitutive model parameters for the use of the proposed model in future finite element analyses to perform more accurate simulations of expanding balloon catheters.

## Introduction

Finite element analysis (FEA) has become a powerful tool for the optimization process of coronary balloon catheters and stents. FEA enables the prediction of the stress/strain response of stents and balloon catheters, and the constituents of the coronary artery to static and dynamic loading. Nevertheless, almost all state-of-the-art FEA of expanding balloon catheter and stent included standard isotropic material models for balloon membranes, in which the mathematical formulation and the material parameters were based on data of unprocessed polymers [1–6]. One exception is presented by Gasser and Holzapfel [7] where a versatile theory of fiber-reinforced materials is used according to [8] in order to mimic the anisotropic material response of angioplasty balloon membranes. However, in their study, the material and structural parameters of the balloon membrane model were not quantified by experimental data but fitted according to the respective inflation characteristic of the analyzed balloon catheter.

Membranes of coronary balloon catheters are mostly produced from polymers like polyethylene (PE), polytetrafluoroethylene (PTFE), polyurethane (PUR), or polyamides (PA). Many groups are known which investigated the morphology and the associated anisotropic material behavior of extrusion-casted films of such polymers by performing uni- or biaxial extension tests, differential scanning calorimetry (DSC), Fourier transform infrared (FTIR) spectroscopy, small-angle X-ray scattering (SAXS), and wide-angle X-ray diffraction (WAXD) [9–12]. However, there is no openly available literature dealing with the study of the multiaxial mechanical properties of processed polymers of balloon catheter membranes.

**Fig 1:**
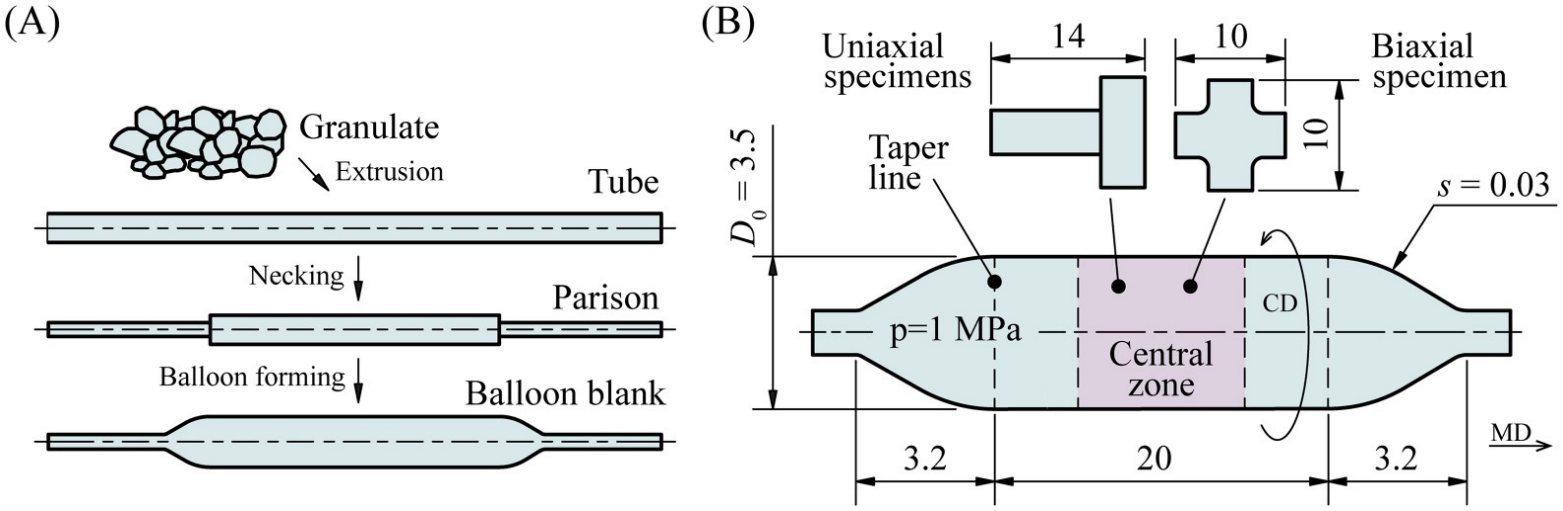
Manufacturing cycle and geometry of the Baroonda SDS balloon catheter. (A) Schematic production cycle of balloon catheter blanks. (B) Illustration of the blank of the Baroonda SDS balloon catheter after blow molding. In the present study, specimens for uniaxial extension, quasi-static and dynamic biaxial testing were taken from the central zone. For location-depending biaxial testing, specimens were also extracted next to the taper lines. All dimensions are in mm and taken from Geith et al. [1]. MD: machine direction; CD: circumferential direction; *D*_0_: outer diameter at the unpressurized state; *s*: thickness of the membrane; *p*: nominal pressure.

Balloon catheter membranes with their typical cylindrical shape and two tapers are produced by the manufacturing technique of balloon-forming, a specific blow-molding technique. The standard procedure was described in detail by Sauerteig and Giese [13], Garramone [14], and Fu et al. [15]. The main production steps, schematically presented in Fig 1A, can be summarized as follows: (i) during extrusion, the dried granular raw material is fed into a screw-driven and heated extruder with a shaping dye and then it is extruded into tubes; (ii) while necking, chilled and cropped tubes get stretched into parisons with necked ends; (iii) in the actual balloon forming process, the parison is inserted into a hollow glass mold and heated up to the specific glass-transition temperature *T*_g_ while being pressurized.

The authors assume that the morphology of balloon catheter membranes changes several times during each of these production steps. Thus, Aggarwal et al. [11] found that the molecular chains of the molten polymer align in flow/machine direction (MD) inside the extruder. However, as soon as the polymer leaves the extruder it cools down and needle-like crystallites start to form. These crystallites proliferate perpendicular to the molecular chains and, therefore, to MD. In the flowing matrix, the crystallites rotate and align parallel to MD, which leads to molecular chains in the crystalline areas pointing perpendicular to MD [12]. With necking most manufactures artificially increase the degree of crystallinity of the ends of the parison, while the central part which is going to be molded into the balloon should remain unchanged. By pressurizing and heating the parison to *T*_g_ during the balloon-forming process, still amorphous molecular chains are free to flow and additionally stretch in circumferential direction (CD) [16]. This can cause an excessive softening of the blank’s membrane in MD. To compensate this effect after the forming pressure is reached and to shape the tapers of the balloon membrane, the blank is additionally stretched in MD during several drawing steps. Thus, the crystallinity of membrane increases. Due to the complexity of the production process, it is plausible that the material response of the final membrane differs from the ones of the raw material through the deliberate reorientation of the crystalline regions and the molecular chains of the polymer. However, it has not yet been investigated whether the manufactured membranes show an isotropic or an anisotropic material behavior.

A polymer that is established in the manufacture of coronary balloon catheter membranes is the semicrystalline polyamide-12 (PA 12). The material behavior of the heated PA 12 during the balloon forming process was experimentally and numerically analyzed by Menary and Armstrong [17] and Fu et al. [15]. However, to the authors’ knowledge, no study is published which documentes the mechanical behavior of finished balloon catheter membranes made of PA 12 or other polymers under uni- or biaxial extension and under realistic testing conditions. Due to the lack of mechanically derived experimental data, the influence of the balloon forming process on the material behavior of PA 12 is not known. The authors suppose, that future FEA of balloon catheter and stent expansion could be significantly improved, by using a suitable material model for polymers with experimentally verified constitutive parameters for PA 12 balloon membranes.

Thus, this study presents a detailed description of the material characteristics of a standard commercial PA 12 balloon catheter for main coronary arteries and proposes a simple micro-structurally motivated constitutive model which is able to mimic the pronounced anisotropic material response observed in the performed experiments. The outcomes finally deliver the essential ingredients for the implementation of realistic balloon catheter material models in FEA. For this purpose, various uni- and biaxial extension tests with quasi-static and dynamic loading scenarios were performed on specimens of a state-of-the-art balloon catheter for coronary interventions. The study also describes how to prepare tiny strip-type as well as cross-shaped specimens and novel clamping techniques for uniaxial- and biaxial tests. Moreover, Cauchy stresses and stretches were computed to analyze the multiaxial viscoelastic features of the PA 12 membranes. Finally, material parameters were identified by fitting the proposed constitutive model to the data set of biaxial extension tests.

## Materials and methods

### Coronary balloon catheter

As in the previous study published by the authors [1], membranes of the Baroonda stent delivery system (SDS, 08BO-3520A, Bavaria Medizin Technologie, Wessling, Germany) were used for all present investigations (see Fig 1B). The balloon membrane of the Baroonda SDS was fabricated from PA 12, also known as ‘Nylon 12’ or under the trademark ‘Grilamid’, featuring a density of *ρ* = 1010 kg/m^3^ and a Poisson’s ratio of *ν* = 0.40. According to the manufacturer, the balloon membrane shows a semi-compliant character, i.e., a limited increase of the diameter past the nominal pressure. The *in vivo* nominal pressure of the Baroonda SDS is *p* = 1 MPa. The cylindrical part of the balloon blank has a length of 20 mm and an outer diameter at the unpressurized state of *D*_0_ = 3.5 mm. The thickness of the membrane is *s* = 30 ± 1 μm.

### Specimen preparation

**Fig 2:**
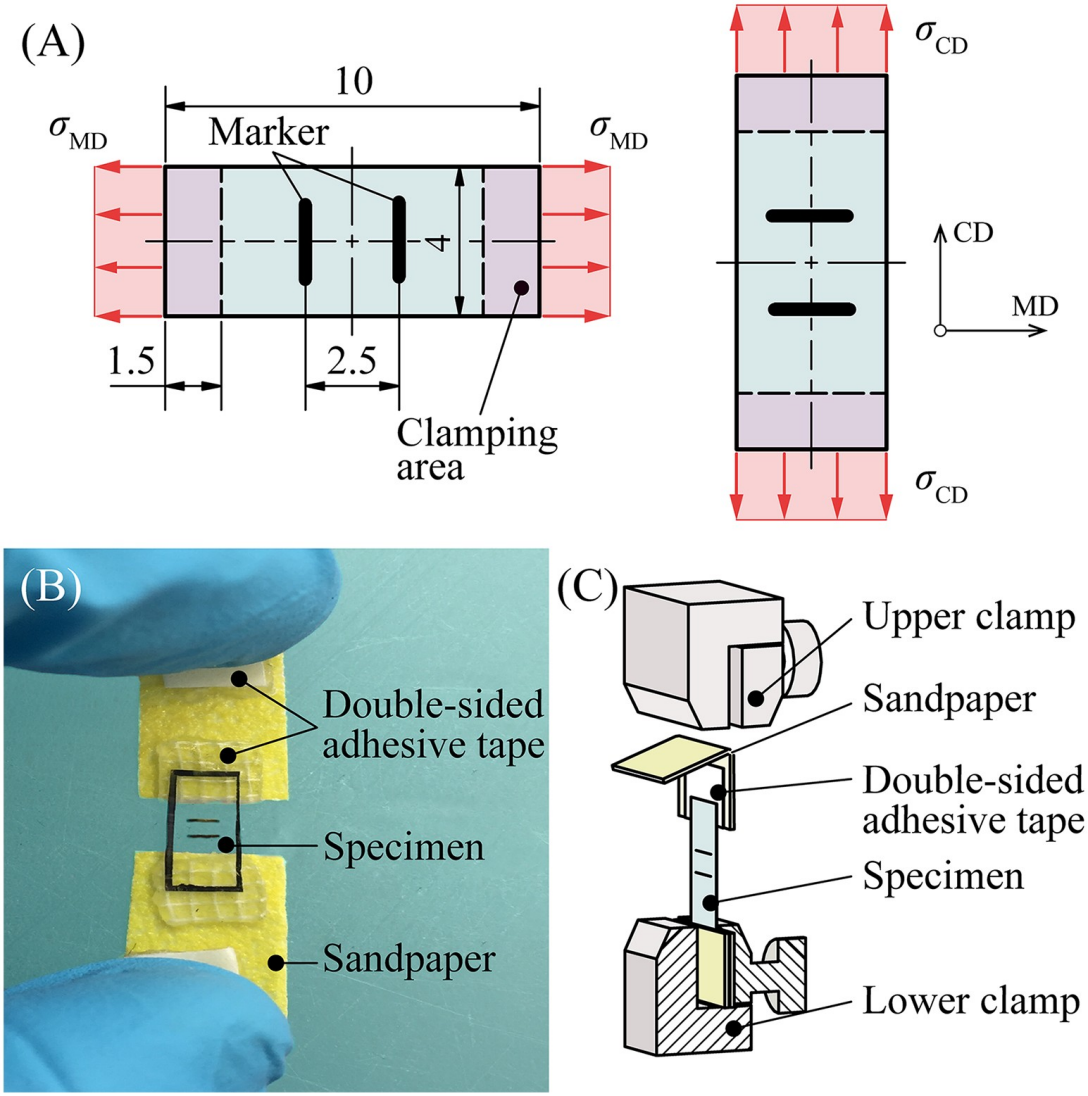
Specimen preparation for uniaxial extension testing. (A) Sketch of a rectangular specimens for uniaxial extension tests cut from the balloon membrane in MD or CD. (B) Photograph of a specimen with double-sided adhesive tape and sandpaper before testing. (C) Two thin markers were added for stretch measurement. Schematic sketch of the clamping method. The rectangular strips were attached to sandpaper with adhesive tape to obtain the required friction between the specimen and the clamps of the uniaxial testing device. All dimensions are in mm. MD: machine direction; CD: circumferential direction; *σ*_MD_: Cauchy stress in MD; *σ*_CD_: Cauchy stress in CD.

**Fig 3:**
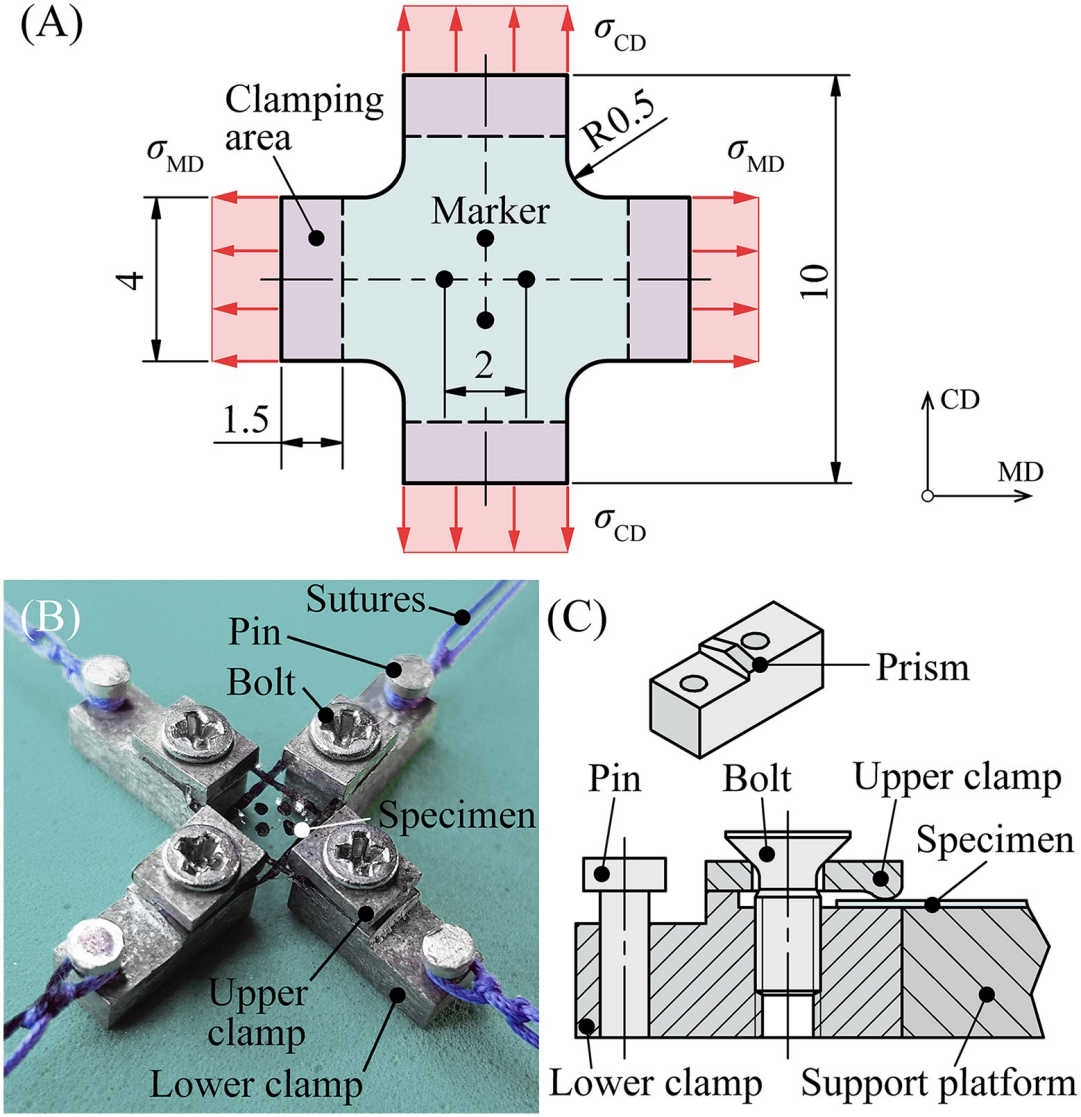
Specimen preparation for biaxial extension testing. (A) Sketch of the cross shape of a specimen for biaxial extension testing. (B) Photograph of the cross-like specimen mounted in the clamping system and surgical sutures. (C) Cross-section of one unit of the clamping system and isometric view of the lower clamp with its prismatic surface. With thin double-sided adhesive tape, the specimens were placed on the support platform, which helps to place all upper and lower clamps symmetrically. All dimensions are in mm. MD: machine direction; CD: circumferential direction; *σ*_MD_: Cauchy stress in MD; *σ*_CD_: Cauchy stress in CD.

For both, uni- and biaxial testing, the tapers of the balloon blanks had to be removed with a scalpel. Afterward, the cylindrical parts of the balloon blanks were pushed over forceps to slice the membrane along MD. The specimen preparation for uni- and biaxial testing differs and can be described as follows:

1. To perform uniaxial extension tests, two samples were excised from the central zone of three membranes, as depicted in Fig 1B—one with its long side parallel to MD and the other with its long side parallel to CD. These rectangular specimens had dimensions of 10 × 4 mm. The long side of the specimens pointed either in MD or in CD of the balloon blank. At the center of every specimen two fine lines with a distance of approximately 2.5 mm between each other were added as markers for subsequent video-extensometer (VE) measurements (Fig 2A).
  To prevent slipping between the specimens and the clamps of the extension testing device, 8 × 4 mm pieces of strong double-sided adhesive tape Doppelband Extrem (45531, UHU, Baden, Germany) had to be attached to 20 × 10 mm pieces of sandpaper Super Easy Cut P120 (65459, SCHULLER Eh’klar, Austria) (Fig 2B). Finally, the bottom surface of each end of the membrane specimens was attached to a piece of sandpaper via previously-applied adhesive tape, and the sandpaper was folded in half to press its upper part with adhesive tape onto the surface of the membrane (Fig 2C).
2. For biaxial testing, specimens with a simple cross shape were found to be the best option after performing several preliminary tests on the tiny balloon membranes. First, square cuts were again taken from the central zone of the balloon membranes (see Fig 1B), which were then cropped into a cross shape. The cross had a dimension of 10 × 10 mm with a flank width of 4 mm (Fig 3A).

To prevent crack formation at the cross edges, a curved transition with a radius of 0.5mm had to be fabricated by using a biopsy punch Integra^™^ Miltex® (3331AA, Integra, Plainsboro, NJ, USA). The center of the cross was marked with four small dots for VE measurments. Due to the small specimen size, a special clamping system had to be designed and manufactured (Fig 3B and 3C). It consists of an upper and a lower clamp, a pin, and a countersunk bolt on each cross side, and a centered support platform. All parts were made of aluminum to minimize the influence of the weight on the measurement results. As a first step, fine double-sided adhesive tape 3M Scotch (6651263, 3M, Saint Paul, MN, USA) had to be placed on the support platform to prevent the specimen from slipping. Next, the cross-shaped specimen was attached to the top surface of the support platform. Here it is important that the inner surface of the membrane faces to the support platform to keep the curling membrane flat. After pushing the lower clamps into the cavities of the platform, the upper clamps were assembled with the countersunk bolts. The top side of the lower clamps is shaped like a prism. Therefore, the back of the upper and lower clamps only touch each other at the peak of that prism. When tightening the bolts, both—upper and lower clamps—align themselves, which exerts a homogeneous clamping pressure along every flank of the cross-shaped specimen. Due to the special shape of the clamps, no sandpaper was needed. Finally, the support platform with the adhesive tape was removed and a thick surgical suture could be tied to every pin for the attachment of the clamping system to the biaxial extension testing device. Both, the specimen geometry and the clamping system were designed to reduce the boundary effects with the aim to obtain a relatively large homogeneous deformation and stress field in the center of the specimen, where the deformations are tracked.

### Uniaxial extension tests

Preliminary mechanical experiments were performed by using the uniaxial extension testing device μ-strain (ME 30-1, Messphysik Materials Testing, Fürstenfeld, Austria) in combination with the 20 N load cell Xforce HP (Zwick Roell, Ulm, Germany) with an accuracy of 0.02% [18]. Every specimen got submerged for 15 min in 37°C warm decalcified water to overcome hygroscopic and thermal changes inside the PA 12. These effects are well known from the literature [19, 20], and might occur during a several minutes lasting coronary intervention. With the results from the VE ME 46-350 (Messphysik Materials Testing, Fürstenfeld, Austria) with a resolution of ±0.15 μm and a sample rate of *f*_s_ = 20 Hz, the specimen stretches λ_MD_ and λ_CD_ were calculated according to

$$\begin{array}{c} \lambda_{\text{MD}}=\frac{l_{\text{MD}}}{L_{0,\text{MD}}}\;\text{and}\;\lambda_{\text{CD}}=\frac{l_{\text{CD}}}{L_{0,\text{CD}}}, \end{array}$$ (1)

where *l*_MD_ and *l*_CD_ are the current deformed lengths and *L*_0,MD_ and *L*_0,CD_ are the reference lengths of the specimen with its long side pointing to MD or CD, respectively. The lengths *L*_0,MD_ and *L*_0,CD_ were measured after the specimen was attached to the device and preloaded with 0.01 N. Before the actual experiment, the specimen was preconditioned. Therefore, several loading and unloading cycles with a testing speed of *v*_test_ = 1 mm/min were performed to a stretch of λ = 1.02. After a maximum of four preconditioning cycles, no more softening was recognized and the stress-stretch behavior of the membrane could be reproduced.

The question of whether preconditioning should be performed prior uni- or biaxial extension testing is controversial. During the preconditioning phase the specimen exhibits a softening effect until the results become reproducible. In fact, most manufacturers expand their balloon catheters several times with the *in vivo* nominal pressure after production to check their functionality. Thus, the authors assume, that the balloon membrane is in a preconditioned state prior to implantation.

For further proceedings, three specimens were stretched with a quasi-static testing velocity of 1 mm/min until rupture. Under the assumption of incompressibility, Cauchy stresses *σ*_MD_ and *σ*_CD_ were calculated as

$$\begin{array}{c} \sigma_{\text{MD}}=\frac{f_{\text{MD}}}{W_{\text{CD}}T}\lambda_{\text{MD}},\;\;\sigma_{\text{CD}}=\frac{f_{\text{CD}}}{W_{\text{MD}}T}\lambda_{\text{CD}}, \end{array}$$ (2)

with *f*_MD_ and *f*_CD_ denoting the current forces, *W*_MD_ = *W*_CD_ = 4 mm and *T* = 0.03 mm are the mean specimen width and the thickness in the reference state, respectively.

### Biaxial extension tests

To determine the multiaxial mechanical response of balloon catheter membranes under typical deformations during coronary interventions, planar biaxial extension tests were carried out. For the tests, a customized biaxial extension testing device described by Sommer et al. [21] with four linear stages, the 50 N load cells U9C (HBM, Darmstadt, Germany) with an accuracy of 0.2% and a position-controlled testing protocol could be used. The stretch measurement was performed with the software Laser Speckle Extensometer (Version 2.23.3.0, Messphysik Materials Testing, Fuerstenfeld, Austria) with a resolution of ±0.15 and a sample rate of *f*_s_ = 20 Hz. Before starting the tests, all specimens were submerged for 15 min in 37°C warm decalcified water. Every side of the cross-shaped specimen was attached to the respective linear stage of the testing rig via the mentioned clamping system and thick surgical sutures. Every suture was placed collinear to the center axis of the respective load cell. The specimen was preloaded in MD and CD to lift the specimen, sutures, and the clamps up to the level of the center lines of the four load cells. Afterward, the VE camera had to be focused onto the specimen and calibrated to enable stretch measurements. Preloading forces of only 0.02 N were vanishingly small in comparison to the maximum forces measured in the proposed study. Therefore, and since stretch measurements were only possible if the specimen was within the focal of the camera, all preloading forces were neglected and set to zero before starting the experiment. In order to observe a possible anisotropic character of the processed PA 12 and to account for its viscoelastic features as well as the location-dependent mechanical behavior of the balloon membrane, three test scenarios were executed as follows:

#### (i) Quasi-static biaxial testing

Three specimens were stretched with a testing velocity of *v*_test_ = 1 mm/min and underwent a systematic sequence of stretch ratios (λ_MD_ − 1): (λ_CD_ − 1) = {1:1, 1:0.75, 0.75:1, 1:0.5, 0.5:1} in MD and CD. Furthermore, the equibiaxial stretch-protocol with an increasing stretch in Δλ = 0.02 steps, starting at λ = 1.02 was performed on three additional specimens. This biaxial testing approach was proven to provide the basic set of material parameters for a subsequent modeling of the in-plane anisotropy [21].

#### (ii) Dynamic biaxial testing

**Fig 4:**
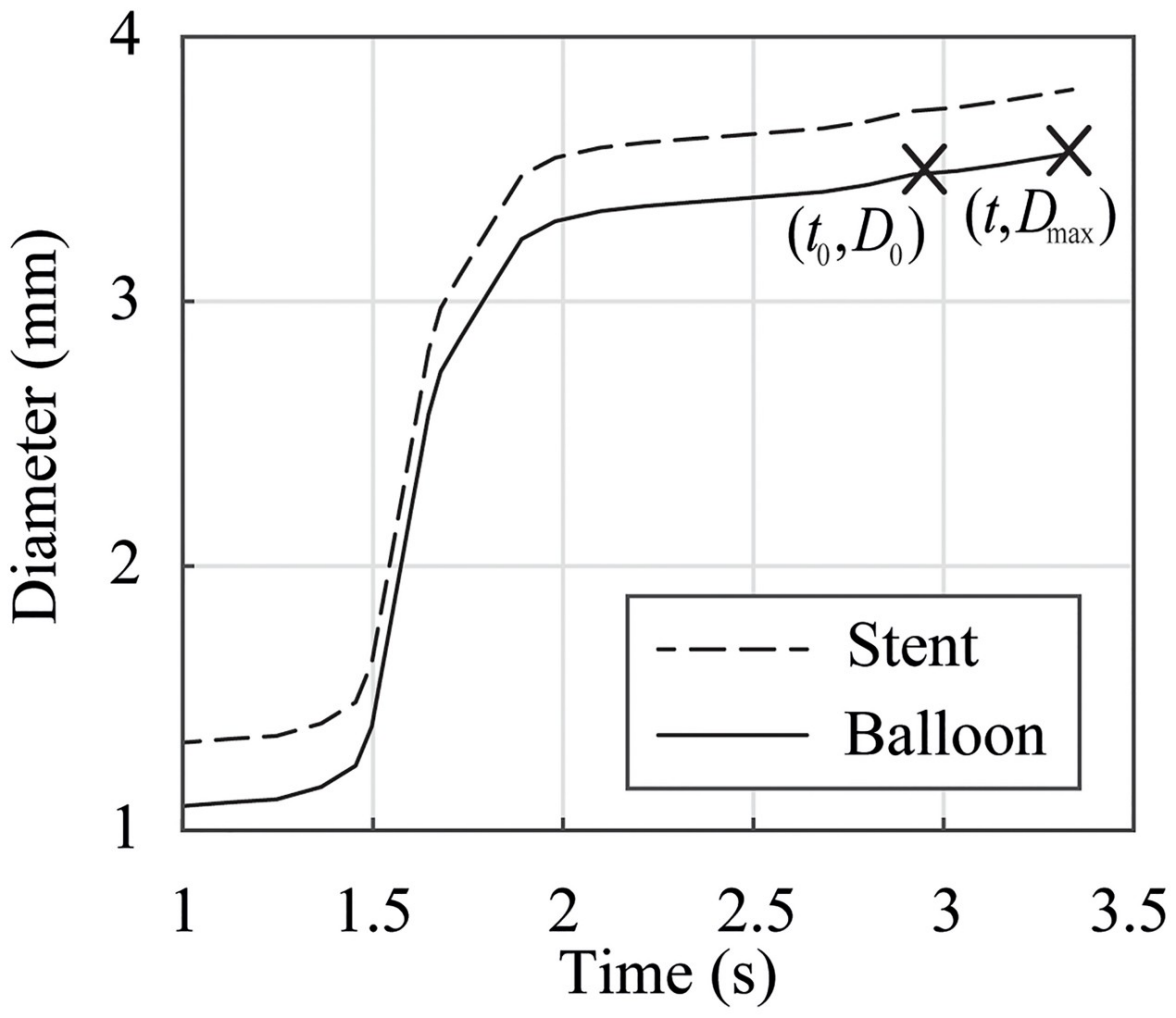
Stent and balloon diameter vs. inflation time. The dashed curve of the stent inflation tests was adapted from Geith et al. [1]. *t*_0_: time at which the balloon reaches its initial diameter; *t*: time at which the balloon reaches its final diameter at nominal pressure; *D*_0_: initial balloon diameter; *D*_max_: diameter at nominal pressure.

To investigate the viscoelastic features of PA 12 under dynamic loading, the tests of (i) had to be repeated with a testing velocity of *v*_test_ = 10 mm/min. In addition, equibiaxial tests on three specimens were performed with the stretch increasing until failure, i.e., the specimens slipped out of the clamps or cracks started to form. An approximation of the actual inflation speed *v*_app_ can be derived as

$$\begin{array}{c} v_{\text{app}}=\frac{\Delta U}{\Delta t}=\frac{\pi (D_{\text{max}}-D_{0})}{t-t_{0}}, \end{array}$$ (3)

where *D*_0_ = 3.5 mm is the diameter of a fully unfolded but yet not stretched balloon catheter membrane at *t*_0_ = 2.95 s in Fig 4. The diameter *D*_0_ equals to the outer diameter of the tested membrane blanks (see Fig 1B). The maximum diameter of the stretched membrane at nominal pressure at *t* = 3.33 s is denoted by *D*_max_. The diameter *D*_max_ of the fully expanded Baroonda SDS balloon catheter can be determined by analyzing the data of the stent inflation tests known from Geith et al. [1] and presented in Fig 4. By measuring the outer diameter of the stent in the fully expanded state under the nominal pressure and by subtracting the stent strut height of *h* = 0.12 mm, the maximum outer diameter is *D*_max_ = 3.57 mm. This leads to an inflation speed of *v*_app_ = 34.72 mm/min and a corresponding stretch of λ_app,CD_ = *D*_max_/*D*_0_ = 1.02 in CD. Despite the fact that *v*_test_ ≪ *v*_app_, a further increase in the testing velocity leads to unreproducible results due to errors in the VE measurements.

#### (iii) Location-depending biaxial testing

One can hypothesize that the preferred orientations of the crystallites and the molecular chains in the taper region differ from the ones in the center of the membrane. To prove this hypothesis, further quasi-static biaxial tests similar to (i) were carried out with specimens that were extracted next to the taper lines of the balloon catheter membrane, as shown in Fig 1B. The data were compared with the results from tests with centered specimens. Only one specimen could be take from every blank due to the short length of the balloon catheter membrane in MD. The authors do not expect different test results for samples taken at different locations along the circumference and, therefore, assume a homogeneous material response along CD.

Because of the limited amount of donated balloon membrane blanks, all biaxial tests—static/dynamic equibiaxial stretch tests, static/dynamic stretch ratio sequences, dynamic equibiaxial rupture tests, and location-depended tests—were only repeated two times. This results in 18 specimens for biaxial testing and additionally six specimens for uniaxial testing. Only tests in which the specimens did not tear at the clamp edges or were pulled out of the clamps were rated as successful. In total 16 biaxial tests and four uniaxial tests failed, which gives a total number of 44 specimens.

Cauchy stresses in MD and CD were computed as

$$\begin{array}{c} \sigma_{\text{MD}}=\frac{f_{1,\text{MD}}+f_{2,\text{MD}}}{2W_{\text{CD}}T}\lambda_{\text{MD}},\;\sigma_{\text{CD}}=\frac{f_{1,\text{CD}}+f_{2,\text{CD}}}{2W_{\text{MD}}T}\lambda_{\text{CD}}, \end{array}$$ (4)

in which *f*_1,MD_, *f*_2,MD_, and *f*_1,CD_, *f*_2,CD_ represent the measured reaction forces and λ_MD_ = *x*_MD_/*X*_MD_, λ_CD_ = *x*_CD_/*X*_CD_ the membrane stretches in MD and CD, respectively, calculated with the marker distances in the actual, loaded (*x*_MD_, *x*_CD_) and the reference (*X*_MD_, *X*_CD_) states. Due to the specimen size, the detection of shear components was neglected and the overall mechanical response of the PA 12 membranes was assumed to be incompressible.

### Material modeling

The PA 12 balloon catheter membrane was modeled as a fiber-reinforced incompressible rubber-like hyperelastic material in which an isotropic matrix material, the amorphous domain, is reinforced by two families of extensible fibers, aligned due to crystallization, rendering the balloon catheter material anisotropic. It is assumed that all fibers of the two fiber families are perfectly aligned, continuously distributed throughout the material and can therefore be described by the two direction vectors **M** and **M**′ in the reference configuration. Hence, the continuum theory of fiber-reinforced materials is the constitutive theory of choice. Briefly, in continuum mechanics a material point **X** of a body in the reference configuration is transformed to a position **x** = ***χ***(**X**) in the deformed configuration by means of the bijective map ***χ***. In addition, the deformation gradient **F** = ∂***χ***(**X**)/∂**X** is introduced, as well as the left Cauchy-Green tensor **b** = **FF**^T^ and the right Cauchy-Green tensor **C** = **F**^T^ **F**. For hyperelastic materials the material response can be quantified in terms of a strain-energy function Ψ, i.e. the recoverable energy stored in the material as it deforms. Hence, the generalized polynomial-type elasticity relations first published by Rivlin and Saunders [22] with a polynomial extension for the anisotropic part were applied. The related strain-energy function Ψ for the fiber-reinforced incompressible material takes on the form

$$\begin{array}{c} \Psi =-\frac{\hat{p}}{2}(I_{3}-1)+\hat{\Psi }\left(I_{1},I_{2},I_{4},I_{6}\right), \end{array}$$ (5)

where the strain-energy function Ψ is defined for the incompressibilty constraint det**C** ≡ 1, $\hat{p}$ is an indeterminate Lagrange multiplier, and the second term is defined as

$$\begin{array}{c} \hat{\Psi }\left(I_{1},I_{2},I_{4},I_{6}\right)=c_{10}\left(I_{1}-3\right)+c_{02}{\left(I_{2}-3\right)}^{2}+c_{11}\left(I_{1}-3\right)\left(I_{2}-3\right)+k_{1}\sum_{i=4,6}{\left(I_{i}-1\right)}^{2}, \end{array}$$ (6)

with *c*_10_, *c*_02_, *c*_11_ and *k*_1_ are material parameters, and *I*_1_, *I*_2_, *I*_4_ and *I*_6_ are four invariants defined by

$$\begin{array}{c} I_{1}=\text{tr}\text{C},\;I_{2}=\frac{1}{2}[(\text{tr}\text{C}{)}^{2}-\text{tr}({\text{C}}^{2})],\;I_{4}=\text{C}:\text{M}⊗\text{M},\;I_{6}=\text{C}:{\text{M}}^{\prime }⊗{\text{M}}^{\prime }. \end{array}$$ (7)

The two invariants *I*_4_ and *I*_6_ denote the square of the fiber stretches in the fiber directions **M** and **M**′, respectively [23]. Thus, the term in Ψ associated with the invariants *I*_4_ and *I*_6_ accounts for the nonlinear stiffening of the fibers with increasing fiber stretch. Both fiber families are modeled as equally strong and are assumed to be symmetric with respect to CD. Hence, the fiber directions [**M**] = [cos *α*, sin *α*, 0]^T^ and [**M**′] = [cos *α*, − sin *α*, 0]^T^ are defined by the angle *α* measured with respect to CD. The constitutive equation for the Cauchy stress tensor ***σ*** is obtained as

$$\begin{array}{c} \sigma =-\hat{p}\text{I}+\left({\hat{\psi }}_{1}+I_{1}{\hat{\psi }}_{2}\right)\text{b}-{\hat{\psi }}_{2}{\text{b}}^{2}+{\hat{\psi }}_{4}\text{m}⊗\text{m}+{\hat{\psi }}_{6}{\text{m}}^{\prime }⊗{\text{m}}^{\prime }, \end{array}$$ (8)

where **m** = **FM** and **m**′ = **FM**′ denote the two direction vectors in the current configuration. For notational simplicity we have introduced the abbreviation ${\hat{\psi }}_{i}=2\partial \hat{\Psi }/\partial I_{i}$, *i* = 1, 2, 4, 6.

For biaxial extension (neglecting shear) the deformation gradient, the left and right Cauchy-Green tensors can be expressed in terms of principal stretches. In the matrix form this reads [**F**] = diag[λ_MD_, λ_CD_, λ_*z*_] and $\left[\text{b}\right]=\text{diag}\left[\lambda_{\text{MD}}^{2},\lambda_{\text{CD}}^{2},\lambda_{z}^{2}\right]=\left[\text{C}\right]$, where λ_*z*_ denotes the stretch orthogonal to the plane spanned by MD and CD. By means of the thin membrane assumption (*σ*_*zz*_ = 0), and by considering the incompressibility constraint (det**C** ≡ 1), the only non-zero components of Cauchy stress tensor ***σ*** are the normal stresses in MD and CD, which read

$$\sigma_{\text{MD}}=-\hat{p}+\left({\hat{\psi }}_{1}+I_{1}{\hat{\psi }}_{2}\right)\lambda_{\text{MD}}^{2}-{\hat{\psi }}_{2}\lambda_{\text{MD}}^{4}+\left({\hat{\psi }}_{4}+{\hat{\psi }}_{6}\right)\lambda_{\text{MD}}^{2}\cos^{2}\alpha ,$$ (9)

$$\sigma_{\text{CD}}=-\hat{p}+\left({\hat{\psi }}_{1}+I_{1}{\hat{\psi }}_{2}\right)\lambda_{\text{CD}}^{2}-{\hat{\psi }}_{2}\lambda_{\text{CD}}^{4}+\left({\hat{\psi }}_{4}+{\hat{\psi }}_{6}\right)\lambda_{\text{CD}}^{2}\sin^{2}\alpha ,$$ (10)

with

$$\begin{array}{c} \hat{p}=\left({\hat{\psi }}_{1}+I_{1}{\hat{\psi }}_{2}\right){\left(\lambda_{\text{MD}}\lambda_{\text{CD}}\right)}^{-2}-{\hat{\psi }}_{2}{\left(\lambda_{\text{MD}}\lambda_{\text{CD}}\right)}^{-4}. \end{array}$$ (11)

The estimation of the material parameters *c*_10_, *c*_02_, *c*_11_, *k*_1_ and *α* was achieved by applying the least-squares method for the nonlinear objective function, which is

$$\begin{array}{c} \phi =\{c_{10},c_{02},c_{11},k_{1},\alpha \}=\underset{\phi }{\text{argmin}}\;\chi^{2}\left(\phi \right), \end{array}$$ (12)

where the objective function is defined as

$$\begin{array}{c} \chi^{2}\left(\phi \right)=\sum_{ij\in \zeta }\frac{\sum_{n=1}^{N_{ij}^{\text{exp}}}(\sigma_{ij}^{n}-{\bar{\sigma }}_{ij}^{n}{)}^{2}}{\text{max}\;({\bar{\sigma }}_{ij}^{n})}, \end{array}$$ (13)

with *ζ* ∈ [MD, CD] are the experimental modes and $N_{ij}^{\text{exp}}$ denotes as the number of of experimental data points for both directions. The analytical results of the stress are denoted by *σ*_*ij*_ and the experimental results by ${\bar{\sigma }}_{ij}$. The normalization is performed in order to weight the data from the two experimental modes equally. With the intention to cover a wide deformation range, three more dynamic equibiaxial extension tests until failure were performed. For the nonlinear data fitting, MATLAB R2019a (The MathWorks, Natick, USA) with the lsqnonlin function was used to estimate the material parameters (*c*_10_, *c*_02_, *c*_11_, *k*_1_, and *α*) by minimizing the least-squares differences between the computational and the experimental data. The quality of the estimation was evaluated by calculating the correlation coefficients $R_{\text{MD}}^{2}$ and $R_{\text{CD}}^{2}$ for both directions according to

$$\begin{array}{c} \begin{array}{c} R_{ij}^{2}=\frac{\sum_{n=1}^{N_{ij}^{\text{exp}}}[(\sigma_{ij}^{n}-\sigma_{ij}^{\text{mean}})({\bar{\sigma }}_{ij}^{n}-{\bar{\sigma }}_{ij}^{\text{mean}})]}{\sqrt{\sum_{n=1}^{N_{ij}^{\text{exp}}}(\sigma_{ij}^{n}-\sigma_{ij}^{\text{mean}}{)}^{2}}\sqrt{\sum_{n=1}^{N_{ij}^{\text{exp}}}({\bar{\sigma }}_{ij}^{n}-{\bar{\sigma }}_{ij}^{\text{mean}}{)}^{2}}} \end{array}\;\text{for}\;\begin{array}{c} ij\in \{\text{MD},\text{CD}\}, \end{array} \end{array}$$ (14)

where $R_{ij}^{2}\in \left[0,1\right]$ ($R_{ij}^{2}=1$ indicates a perfect estimation of the model parameter), and $\sigma_{ij}^{\text{mean}}$, ${\bar{\sigma }}_{ij}^{\text{mean}}$ are mean stresses, while $\sigma_{ij}^{n}$, ${\bar{\sigma }}_{ij}^{n}$ are the stresses at any data point predicted by the model and calculated from the experimental data, respectively. The root-mean-square error (RMSE) was calculated by

$$\begin{array}{c} \text{RMSE}=\frac{\sqrt{\frac{\chi^{2}(\phi )}{\sum_{ij\in \xi }N_{ij}^{\text{exp}}-q}}}{\sum_{ij\in \xi }{\bar{\sigma }}_{ij}^{\text{mean}}}, \end{array}$$ (15)

in which *q* is the number of parameters in *ϕ*, compare with Holzapfel *et al*. [24] and Schulze-Bauer *et al*. [25].

Robustness of the parameter estimation was ensured by using: (i) a variety of different loading conditions (i.e. stretch ratios) to make sure that the hyperelastic material model is able to predict correct stress values under all conditions of interest; and (ii) different roots for the initial guesses of the fitting procedure. A minimum of six minimization cycles ensured that best-fit parameters are independent of the initial guesses. Hence, the authors focus here more on the robustness of the entire model prediction than on the individual parameters. Nevertheless, physical relevance of the parameters has to be ensured, e.g. by restricting the search space [26].

Although the authors assume that PA 12 is highly viscoelastic, a respective model feature has not been implemented. Similar to many FEA based studies who describe the maximum stresses in stents and catheters during expansion [1–6], only the loading curve is of interest to the authors to be able to investigate the maximum stresses in vascular tissue in future work.

## Results

### Uniaxial mechanical response

**Fig 5:**
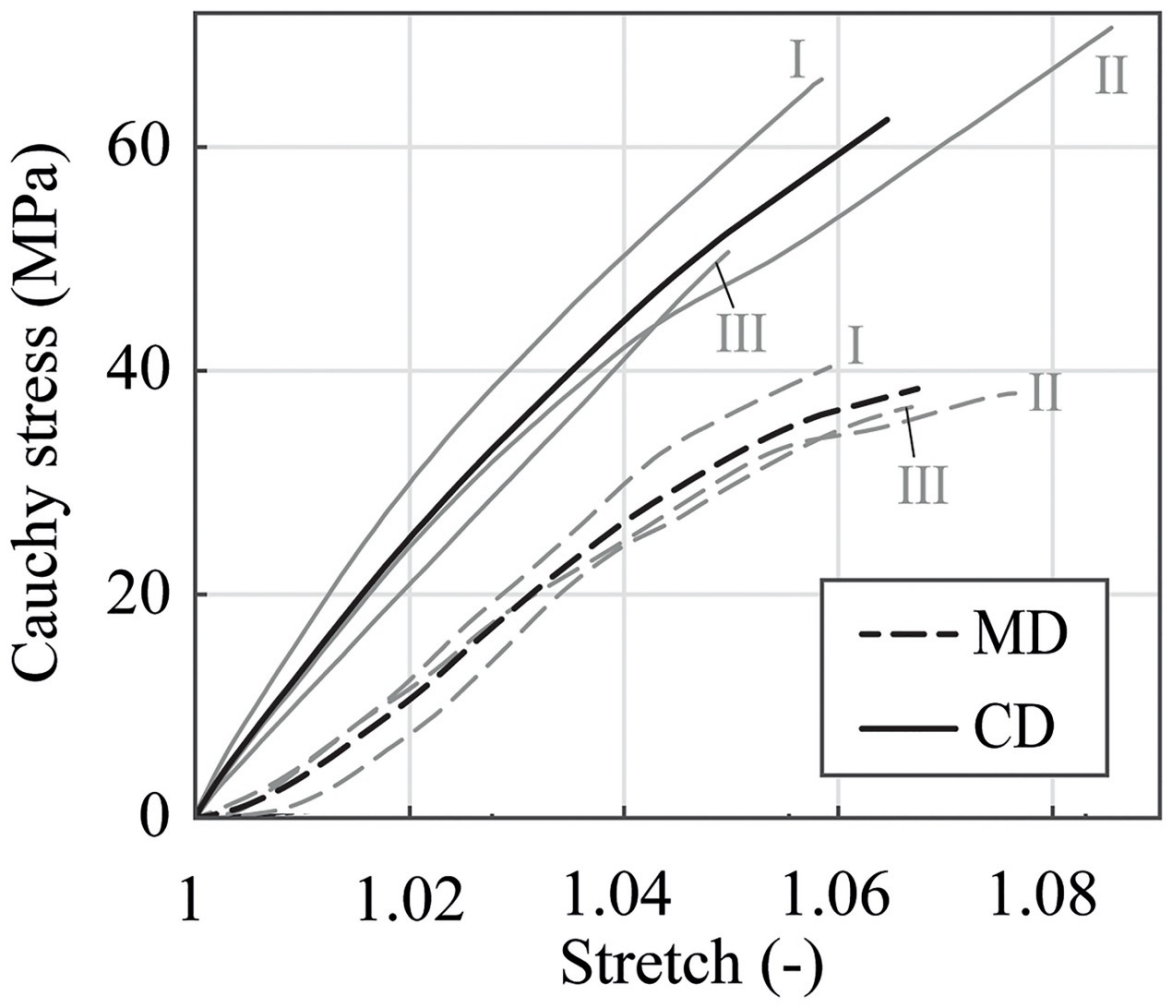
Uniaxial Cauchy stress-stretch relationship in MD and CD. The diagram shows the results of respectively three specimens (I, II, III). The bold solid and bold dashed curves represent the corresponding median curves.

A representative Cauchy stress-stretch response of the PA 12 balloon membrane during preliminary uniaxial rupture tests is presented in Fig 5. The tests reveal a nonlinear and anisotropic stress-stretch response of PA 12 in MD and CD. The membrane shows a pronounced stiffer response for small and large stretches in CD. In MD, the specimens are very ductile, especially at minimal and very large stretches, while an increase in the stiffness is detectable at medium stretches. The median of the Cauchy stress in MD at λ = 1.02 was computed to be ${\tilde{\sigma }}_{\text{MD}}=8.4$ MPa, which is 40.2% of the corresponding Cauchy stress at the equal stretch λ_app,CD_ at nominal pressure in CD with ${\tilde{\sigma }}_{\text{CD}}\left(\lambda_{\text{app},\text{CD}}\right)=20.9$ MPa. The median of the ultimate tensile stress at rupture in MD was calculated to be ${\tilde{\sigma }}_{\text{MD},\text{max}}=32.0$ MPa, which is 61.4% of the ultimate tensile stress in CD with ${\tilde{\sigma }}_{\text{CD},\text{max}}=52.1$ MPa. The corresponding ultimate tensile stretches were determined to be ${\tilde{\lambda }}_{\text{MD},\text{max}}=1.0675$ and ${\tilde{\lambda }}_{\text{CD},\text{max}}=1.0645$.

### Biaxial mechanical response

**Fig 6:**
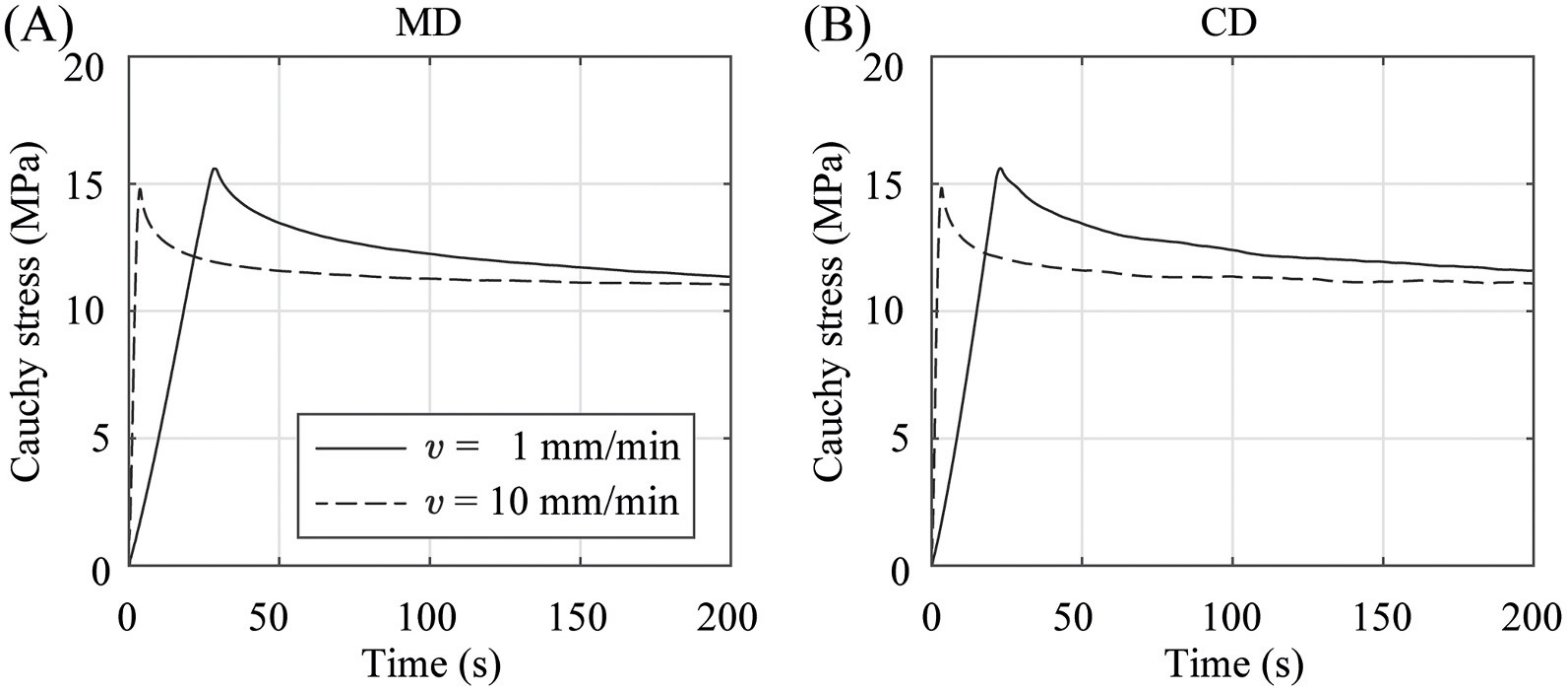
Representative quasi-static (dashed curve) and dynamic (solid curve) equibiaxial relaxation behavior. Normal Cauchy stress (A) in MD and (B) CD under a stretch of λ = 1.02 as a function of time.

The graphs in Fig 6 present the characteristic equibiaxial relaxation behavior (Cauchy stress vs. time) of the PA 12 membranes under quasi-static (*v*_test_ = 1 mm/min) and dynamic loading (*v*_test_ = 10 mm/min). Similar graphs for both, MD and CD, indicate an isotropic material response. Upon closer inspection of the graph during the first seconds, it shows a nonlinear increase over time followed by an immediate drop and a gradual decrease of the Cauchy stresses of approximately 25% after the linear stages of the testing device stopped when the stretch of λ = 1.02 ≙ λ_app,CD_ was reached and held in position for *t* = 200 s. This is a clear evidence of the viscoelastic nature of PA 12. In comparison, the results of the dynamic equibiaxial relaxation tests illustrated in Fig 6 indicate a faster material response, however, without any strain-rate depending stiffening. No significant differences in the maximum Cauchy stresses and the Cauchy stresses in the relaxed state were found between quasi-static and dynamic testing. Nevertheless, the subsequent drop of the Cauchy stresses happened faster during dynamic testing.

**Fig 7:**
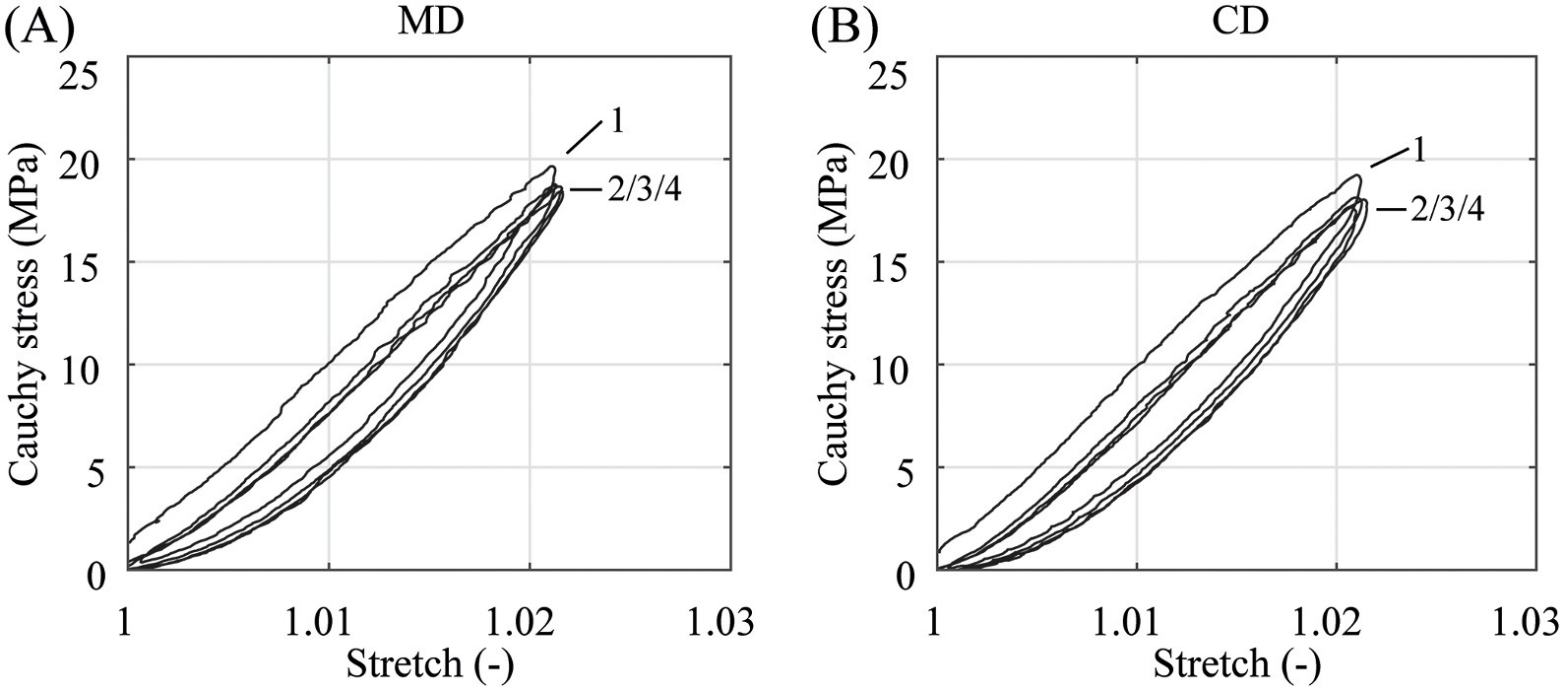
Representative quasi-static preconditioning behavior. (A) in MD and (B) CD at a stretch of λ = 1.02 and under a testing velocity of *v* = 1 mm/min. Due to softening, cycle 1 induced a small offset and shifted cycles 2–4 to the right.

**Fig 8:**
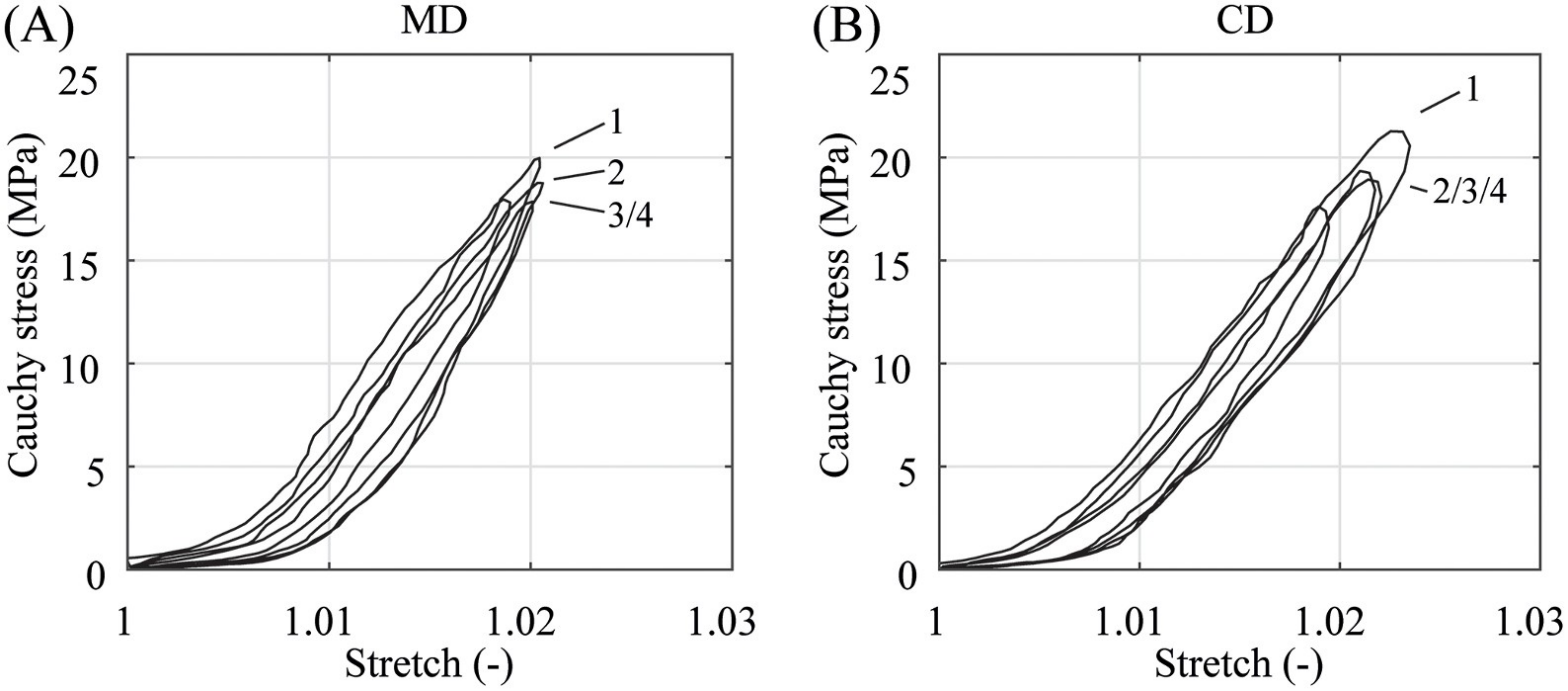
Representative dynamic preconditioning behavior. (A) in MD and (B) CD at a stretch of λ = 1.02 and under a testing velocity of *v* = 10 mm/min. Due to softening, cycle 1 induced a small offset and shifted cycles 2–4 to the right.

In the preconditioning, where the specimens were equibiaxially deformed under quasi-static and dynamic conditions with a small stretch of only λ = 1.02 ≙ λ_app,CD_, the membranes show no significant difference in the material behavior, see Figs 7 and 8. The illustrated stress-stretch relationship is almost identical apart from a more compliant initial material response during dynamic preconditioning. It appears that kinetic effects and the predominant direction of the polymer chains have hardly any influence on biaxially deformed PA 12 balloon catheter membranes under nominal pressure. This is in contradiction to the measurement data from preliminary uniaxial tests, where the data in Fig 5 suggest a distinctive anisotropic material characteristic even in a low stretch range (λ ≤ 1.02). In both, the quasi-static and dynamic testing, the PA 12 membranes show no more softening after the third loading and unloading cycle in both direction (Figs 7 and 8). However, the first preconditioning cycle induced an offset and, therefore, shifted the following curves to the right. This offset was small during dynamic preconditioning. After the forth preconditioning cycle, the current marker distance at force zero was defined as the reference marker distance, denoting λ = 1, for all subsequent measurement cycles.

**Fig 9:**
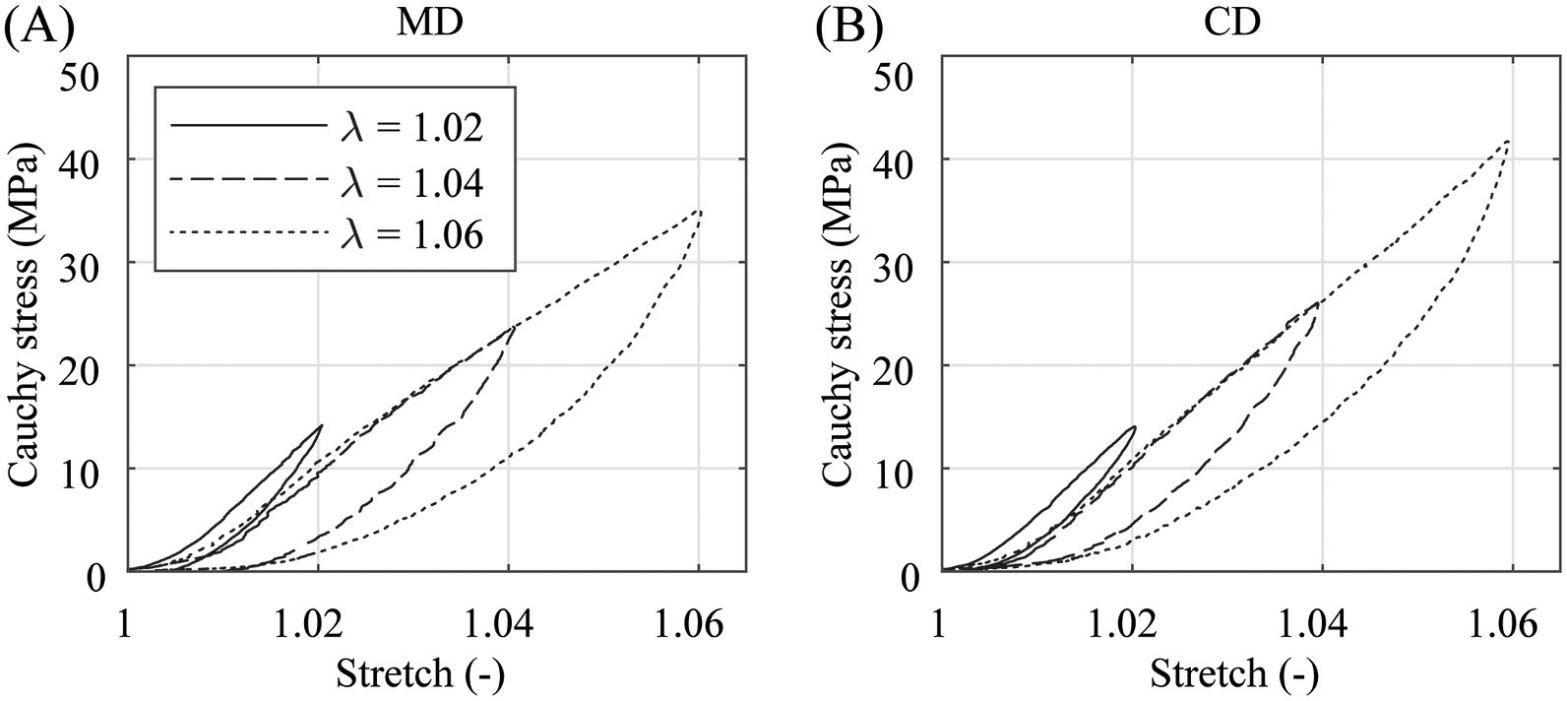
Representative quasi-static equibiaxial Cauchy stress-stretch behavior. (A) in MD and (B) CD under various stretch levels ranging from 1.02 to rupture in 0.02 stretch increments and a testing velocity of *v* = 1 mm/min.

**Fig 10:**
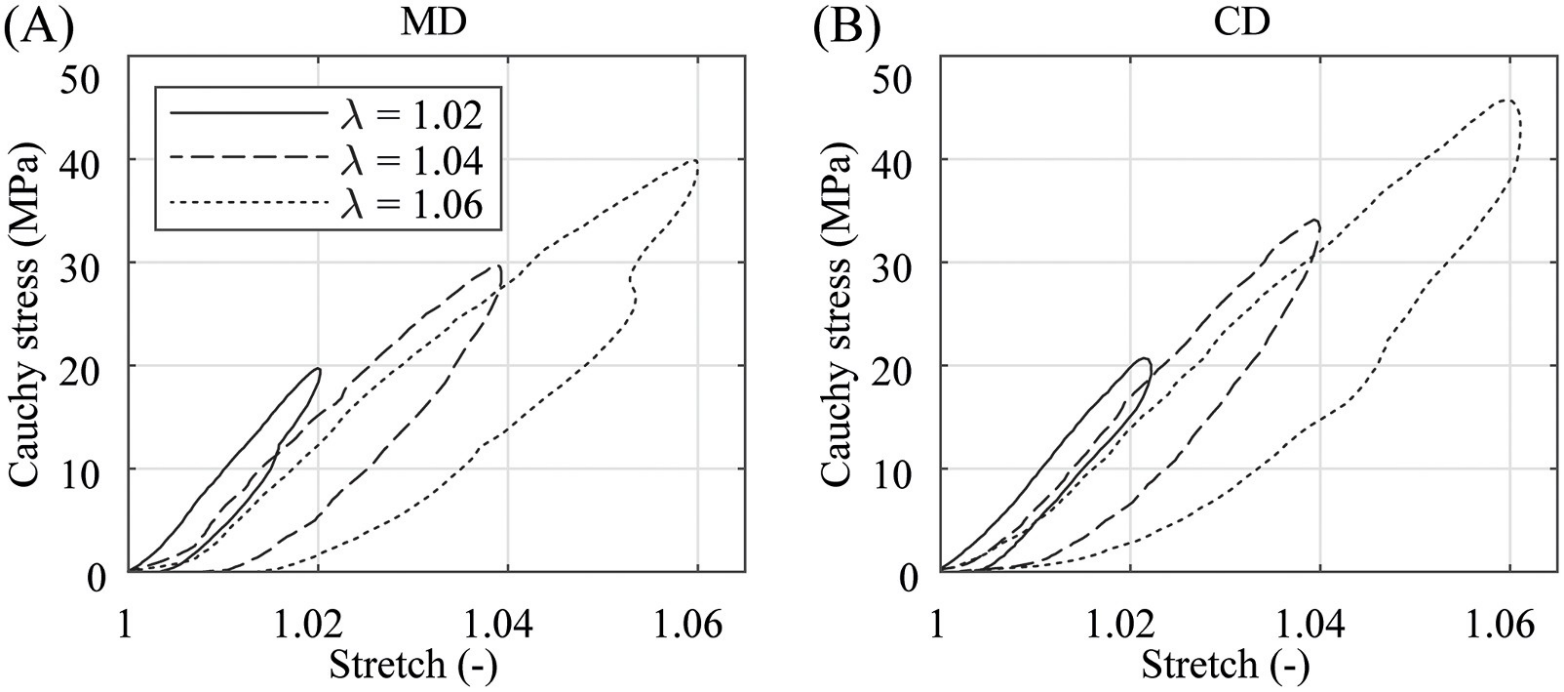
Representative dynamic equibiaxial Cauchy stress-stretch behavior. (A) in MD and (B) CD under various stretch levels ranging from 1.02 to rupture in 0.02 stretch increments and a testing velocity of *v* = 10 mm/min.

The graphs in Figs 9 and 10 enable a comparison of quasi-statically and dynamically loaded PA 12 membranes under different equibiaxial stretch levels ranging from smaller stretches λ = 1.02, to medium (λ = 1.04) and larger stretches (λ = 1.06). While the material response of all specimens of both test modes is of an isotropic nature for smaller stretches only, the data reveal an increasingly significant anisotropic characteristic as soon as the stretches exceed λ = 1.02. The maximal Cauchy stresses are also higher in the dynamically loaded specimens. Another indicator for the viscoelastic material behavior of PA 12 is the presence of forming hysteresis loops between the loading and unloading during preconditioning and equibiaxial measurement cycles. While the hysteresis loops are rather small after small stretches, they show a much more pronounced shape after medium (λ = 1.04) and larger stretches (λ = 1.06). However, the development of the hysteresis loops with increasing stretches is equal for quasi-statically and dynamically tested PA 12 membranes. Noticeable is only the slightly curved peaks of the graphs in Fig 10 in comparison to the sharp peaks in Fig 9. This is based on the inertia of the material response during fast loading changes, which can be attributed to the viscoelasticity of the material or to the time delay of the VE. One may assume that the loading curves in Figs 9 and 10 should be collinear. However, this is not true for smaller stretches in the quasi-static tests and for the whole stretch range in the dynamic tests. The authors suppose that micro-damage or rearrangement of the molecular chains occurred during every equibiaxial loading cycle, which are more pronounced during dynamical testing.

**Fig 11:**
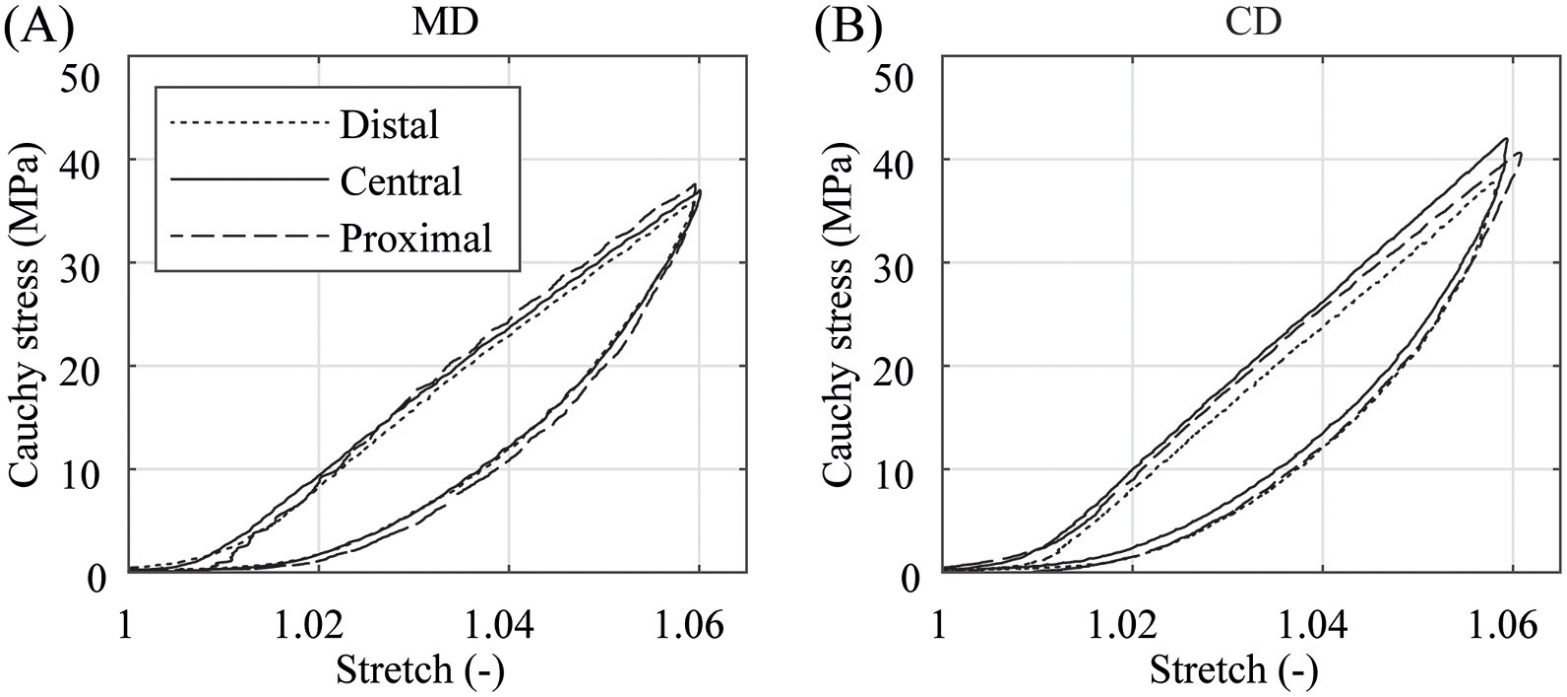
Representative comparison of the Cauchy stress-stretch behavior of the distal, central, and proximal parts of PA 12 balloon catheter membranes. (A) in MD and (B) CD under a stretch of λ = 1.04 and a testing velocity of *v* = 1 mm/min.

A representative comparison of the Cauchy stress-stretch relationship of specimens taken from different locations of the cylindrical part of the balloon catheter membrane is shown in Fig 11. No significant deviation of the graphs in the respective directions had been found. It can, therefore, be assumed that PA 12 shows a homogeneous material behavior along the cylindrical part in MD of the balloon catheter membrane.

**Fig 12:**
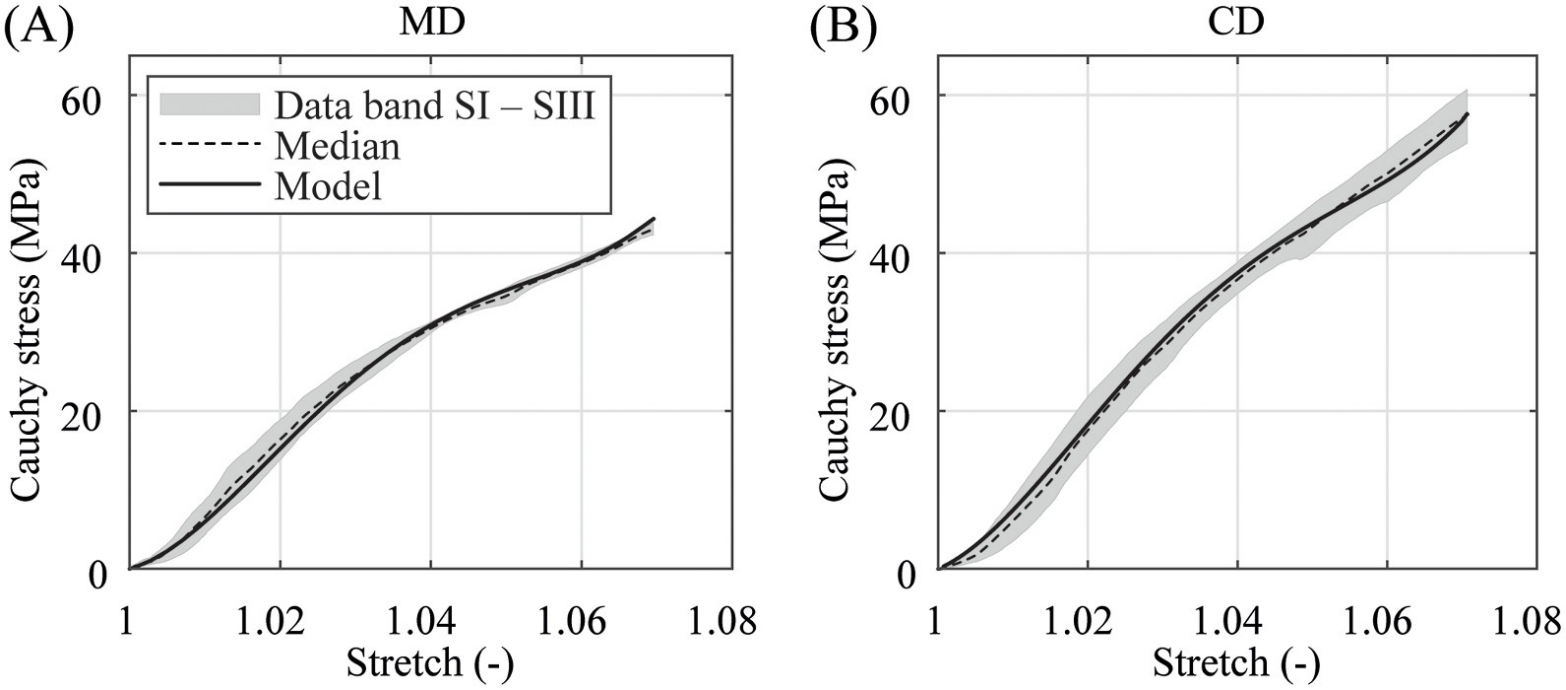
Cauchy stress-stretch behavior until failure and material model response. (A) in MD and (B) CD under a testing velocity of *v* = 10 mm/min. All values of the tested Specimens SI–SIII are within the gray data band.

The material responses of three specimens until failure and their medians in MD and CD are illustrated in Fig 12. Again, as during cyclic loading, the material shows an isotropic response until a stretch of λ ≤ 1.02 is reached and then manifests a distinctive anisotropic characteristic. The median of the normal Cauchy stress at nominal pressure λ = 1.02 ≙ λ_app,CD_ was computed to be ${\tilde{\sigma }}_{\text{MD}}=16.7$ MPa in MD, which is 92.3% of the corresponding Cauchy stress ${\tilde{\sigma }}_{\text{CD}}\left(\lambda_{\text{app}}\right)=18.1$ MPa in CD. At λ = 1.04 the difference increases (${\tilde{\sigma }}_{\text{MD}}=30.4$ MPa, ${\tilde{\sigma }}_{\text{CD}}=36.9$ MPa, pct = 82.4%), becomes more significant at λ = 1.06 (${\tilde{\sigma }}_{\text{MD}}=38.7$ MPa, ${\tilde{\sigma }}_{\text{CD}}=50.1$ MPa, pct = 77.3%), and finally peaks at failure (${\tilde{\sigma }}_{MD,\text{fail}}=44.5$ MPa, ${\tilde{\sigma }}_{CD,\text{fail}}=58.2$ MPa, pct = 76.5%). The corresponding median of the failure stretch ${\tilde{\lambda }}_{\text{fail}}∼1.07$ is 3.5 times higher than the stretch under nominal pressure λ_app_ = 1.02.

Nevertheless, all graphs generated from experimental data in Figs 9, 10 and 12 feature a short exponential increase in the Cauchy stresses under small stretches. At around λ ∼ 1.02 and after a further inflection point at λ ∼ 1.04 the graphs are polynomial; nearly linear until failure. Thus, the balloon membranes show the typical stress vs. stretch progression for polyamides known from the literature [27–29].

### Model parameters

**Fig 13:**
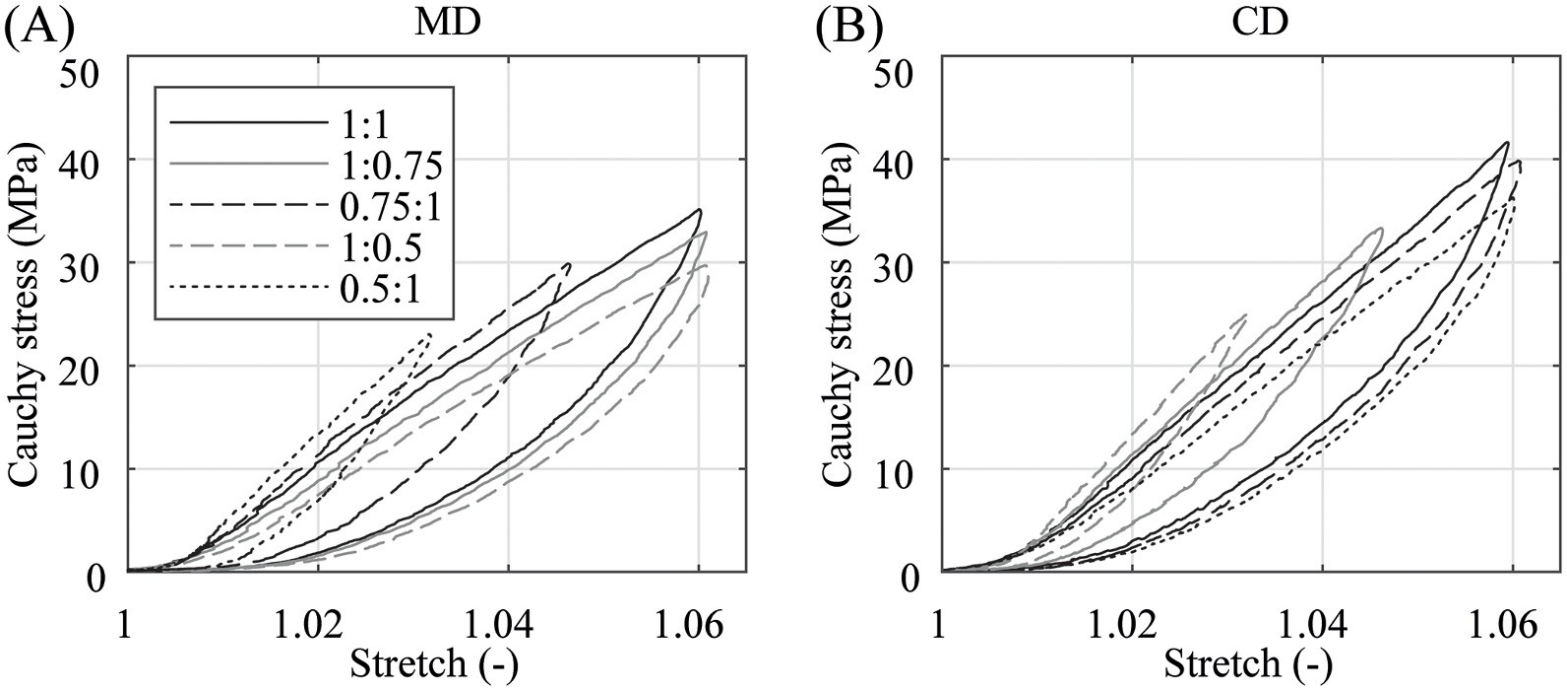
Representative quasi-static biaxial Cauchy stress-stretch behavior for different stretch ratios. (A) in MD and (B) CD with a testing velocity of *v* = 1 mm/min. Stretch ratios λ_MD_: λ_CD_ = {1:1, 1:0.75, 0.75:1, 1:0.5, 0.5:1}.

**Fig 14:**
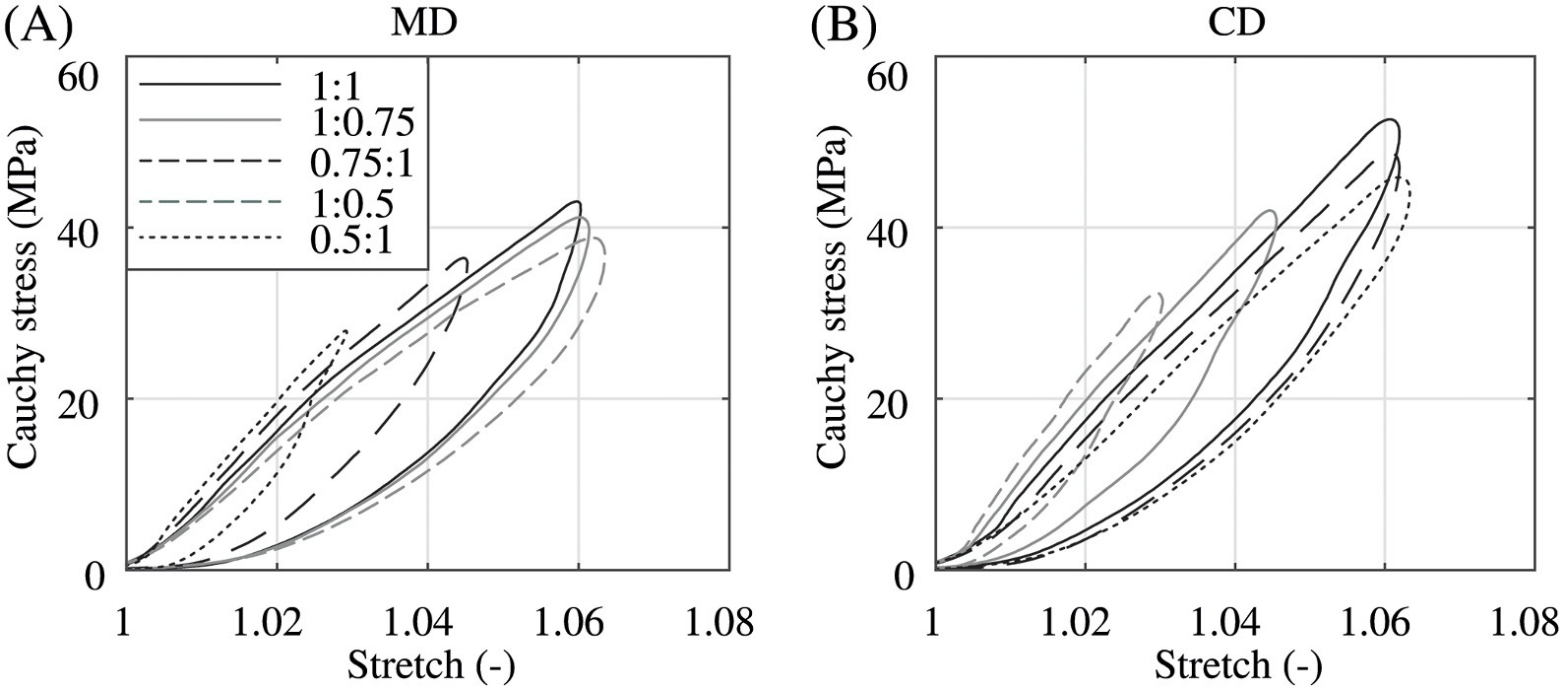
Representative dynamic biaxial Cauchy stress-stretch behavior for different stretch ratios. (A) in MD and (B) CD with a testing velocity of *v* = 10 mm/min. Stretch ratios λ_MD_: λ_CD_ = {1:1, 1:0.75, 0.75:1, 1:0.5, 0.5:1}.

The graphs in Fig 12 illustrate the comparison between the model fit and the experimental data. The proposed model was able to give a very satisfying representation of the dynamic equibiaxial behavior for the whole stretch range ending at a stretch of λ ∼ 1.07. The model parameters and the associated median and the deviations between the fit and the experimental data of all three test specimens *SI–SIII* are shared in Table 1. For future advanced modeling approaches, e.g., the mathematical description of the viscoelastic features, the authors want to share a representative biaxial tensile response of the PA 12 balloon catheter membrane at different stretch ratios between MD and CD under quasi-static and dynamic loading represented in Figs 13 and 14, respectively.

**Table 1:** Constitutive model parameters, squared pearson’s coefficients *R*_CD_, *R*_CD_, and the root-mean-square error RSME for the proposed model (5). All results were obtained by fitting the hyperelastic strain-energy function to the median of the experimental equibiaxial data of the tested PA 12 balloon catheter membranes.

	Constitutive parameters					$R_{\text{CD}}^{2}$ (−)	$R_{\text{MD}}^{2}$ (−)	RMSE (−)
	*c*_10_ (MPa)	*c*_02_ (MPa)	*c*_11_ (MPa)	*k*_1_ (MPa)	*α* (°)			
Median	−1791	28736	−36242	2770	44.77	0.9995	0.9992	0.02

## Discussion

Balloon catheter membranes significantly influence the expansion behavior of stents. Despite the well-known fact that the molecular chains of extruded polymers exhibit a preferred direction, current studies [1–6] focusing on the optimization of stent designs and stent delivery systems by the help of the FEA, using almost exclusively isotropic material models for balloon catheter membranes. This work shows, however, that the decision to use an isotropic or anisotropic material model must be carefully considered.

This statement is based on the key finding of this work, in which the material behavior of the PA 12 balloon catheter membrane shows a stiffer response to loading in CD, as illustrated in Figs 9, 10, 13 and 14. The stiffer response might be the result of the predominant orientation of molecular chains in CD in extruded and stretched polymers, as described in several studies [10–12]. By examining the results from uniaxial extension tests depicted in Fig 5, this effect is even noticeable within the whole loading range. On the contrary, the stress-stretch relationship of specimens under small equibiaxial stretches is almost identical but starts to differ at stretches λ above 1.02. The authors assume that the biaxial mechanical response of PA 12 under small stretches is highly influenced by the coupling between the polymer chains (‘Poisson effect’). Stretches arising in CD will certainly be accompanied by stretches in MD. The influence of this effect probably decreases with higher stretches and an anisotropic material behavior emerges. On average, the specimens showed a slightly softer behavior in uniaxial than in biaxial extension. This conforms with the expectation that the ‘Poisson Effect’ would favor a stiffer reaction in biaxial extension. Compared to other tested polymers [30, 31] and injection molded PA 12 [20], the processed balloon catheter membranes reveal a very stiff response even at small stretches. This is an indicator of the semi-compliant characteristic of the Baroonda SDS membrane which was achieved through a tailored balloon-forming process.

The induced stresses in the PA 12 membranes were found to be higher during dynamic equibiaxial testing above nominal pressure. The increase in the maximum stresses due to a higher strain-rate conforms with the findings of McFerran et al. [20]. In the relaxation tests, the difference between the maximum stresses and the stresses after 200 s did not increase with a higher testing speed. The effect of strain-rate dependence only pronounces at larger stretches. Thus, no significant influence of the strain-rate between quasi-static and dynamic testing modes at small stretches could be detected.

Furthermore, a characteristic of the viscoelastic nature of balloon-molded PA 12 membranes was observed. The results from quasi-static and dynamic relaxation tests reflect the typical time-dependency of the strain-recovery inside initially loaded polymers if compared with Meyer et al. [30]. A further indicator for the typical viscoelastic polymer-like response is the pronounced hysteresis in the equibiaxial tests. The differences in the loading and unloading curves become more significant in the dynamic testing mode. The authors assume that the viscoelastic response of PA 12 is again associated with the rearrangement of molecular chains in the amorphous regions, as described for polypropylene by Coulon et al. [32].

Preconditioning of PA 12 balloon catheter specimens should be taken into account for extension tests as the balloon catheters usually get pressurized several times after manufacturing to test its functionality. However, only a minor effect of the cyclic loading during preconditioning on the mechanical response at small stretches was found. The difference between the first and the last preconditioning cycle seems to be the result of early stretches based on viscoplastic effects. The viscoplasticity of polymers at small stretches can usually be explained with rearrangement mechanisms in the amorphous regions and micro-damage mechanisms. Similar to the tests of Meyer et al. [30], the first loading cycle in CD and MD of the quasi-static and dynamic preconditioning shows a large initial hysteresis loop. The hysteresis loops of the following loading cycles become smaller and the maximum stresses slightly drop.

No differences in the stress-stretch behavior along the cylindrical part of the Baroonda SDS membrane was found. Biaxial extension tests with specimens taken from the distal, central, and proximal part of the membrane show similar results.

The presented clamping system of the VE-based biaxial extension tests was found to be suitable for small specimens with a cross shape. By choosing to manufacture the clamps from aluminum and the small dimensions of the clamps, the influence of the clamps on the measurement results could be reduced to a negligible amount. Due to the novel design of the clamps, a homogeneous clamping pressure distribution along every flank of the specimens can be assured. Besides, the support platform guaranteed the symmetrical arrangement of all four clamps. The sample design also leaves enough space to place four markers for VE-measurements. Furthermore, biaxial testing better represents the multiaxial loading case during percutaneous coronary intervention than uniaxial testing. Thus, biaxial testing provides an adequate setting for the proposed modeling approach.

As mentioned above, standard PA 12 balloon catheter membranes show an almost isotropic behavior at small stretches when biaxially loaded. Small stretches occur within the balloon pressure range between zero to nominal pressure of the Baroonda SDS balloon catheter. In this case it would be reasonable to use an isotropic material model that can numerically describe the material response to small stretches based on the experimental data of this work. If the balloon catheter gets pressurized above the nominal pressure, the anistropic character of the membrane clearly emerges. In this case, the authors suggest to continue with the modeling approach presented in this study. Inspired by the work of Rivlin and Saunders [22], the modified version of the polynomial model used shows overall a very satisfying fit of the loading curve of dynamic tested PA 12 membranes. Note, however, that the proposed material model is a purely elastic model, and hence it does not account for material damage. Manufacturers and scientists have to deliberate how accurate FEA results need to be in simulations with balloon catheters pressurized above the nominal pressure to meaningfully contribute to the optimization process of balloon catheters and stents with respect to computational expenses. It should be noted that the internal balloon pressure most likely exceeds the nominal pressure several times during the coronary treatment. The reason for this could be the geometry or material properties of the vital part of the artery, the stenosis, or the stent, and accidents or unwary performance.

Even though all presented methods appear to be robust to quantify the stretches of PA 12 balloon catheter membranes and to model them accordingly, this study has some limitations. The presented model, and the constitutive parameters, account only for the biaxial material behavior of standard PA 12 balloon catheter membranes for main coronary interventions, i.e., for balloon catheters with a thickness of approximately 30. Although almost all manufacturing processes of balloon catheter membranes are standardized, varying the manufacturing parameters can influence the morphology of PA 12 and, therefore, its mechanical response. In contrast to biaxial testing, where the sample is in direct contact with the rigid clamp, the elastic adhesive tape and the sandpaper may contribute to the measured material response. It is possible that the adhesive tape and the sandpaper slightly falsify the measured material response. For the biaxial testing modes, the influence of shear stresses was not observed due to the small size of the specimens. Thus, with the used VE-based method, it can only be assumed that the stress-stretch distribution in the field of interest is homogeneous and shear deformation have no major influence on the normal stresses. Furthermore, the realistic strain-rate could not be fully achieved with the present biaxial extension testing device. Due to the limited amount of donated balloon catheter blanks and the extent of preliminary testing, the number of the final test series is small. Further biaxial extension tests might be necessary to substantiate the statistical validity of the findings and to observe the influence of shear stresses on the results. In addition, the modeling approach in this study does not incorporate the observed viscoelastic nature of PA 12; incorporation of a viscoelastic model for this type of anisotropic material at finite strains can be achieved [24]. To the authors’ opinion, vascular injuries, and possible damage to the balloon or the stent occur mainly during the expansion phase of the implantation. Therefore, only the modeling of the dynamic loading behavior was of interest. However, the complex mathematical description of the strain-dependency and the hysteresis formation of the balloon membranes should be the matter of further studies.

## Conclusion

In conclusion, the cylindrical part of the PA 12 membranes of the Baroonda SDS balloon catheter for main coronary arteries can be characterized as homogeneous, semi-compliant, and viscoelastic, which shows a nonlinear isotropic material response at nominal balloon pressure when being loaded biaxially. However, above the nominal pressure, anisotropy, and strain-rate dependency of the material behavior emerges significantly with increasing stresses.

To the authors’ knowledge, this study presents the first set of experimental data of the mechanical response of standard PA 12 balloon catheter membranes for main coronary arteries. In uni- and biaxial extension tests, a stretch-driven protocol was used to simulate the loading scenarios of balloon catheter membranes expanding with an inner pressure ranging from zero to the supra-nominal level. Furthermore, novel methods are described to carry out quasi-static and dynamic extension tests on tiny planar specimens taken from balloon catheter membranes for the first time. The study provides a modeling approach, which uses a generalized polynomial-type elasticity relation together with a polynomial extension for the anisotropic part. This model can reproduce the biaxial behavior of the tested PA 12 membranes very well.

Thus, the authors assume that even a FEA simulating the expansion of PA 12 balloon catheter membranes with nominal pressure—e.g., the authors’ previous work [1]—may benefit by implementing the proposed model. The isotropic material response of the balloon catheter in such a FEA would finally be based on experimental data from extension tests performed on actual PA 12 membranes. However, it cannot be excluded that the stresses inside the balloon membrane reach a supra-nominal level, e.g., due to incorrect handling of the catheter or due to unfavorable contact situations with stents or stenoses. Therefore, for FEA, in which the consequences of an over-expanded membrane on the balloon catheter, stent, and the artery due to supra-nominal pressurization are of interest, the authors advise the use of the proposed material model. Furthermore, experimental data, specifying the material response of PA 12 membranes at different stretch ratios, and rates are provided in this article. Based on these data, the proposed model could be improved by incorporating the viscoelastic features of the tested PA 12 membranes.
